# Feature analysis of cell nuclear chromatin distribution in support of cervical cytology

**DOI:** 10.1117/1.JMI.4.4.047501

**Published:** 2017-10-17

**Authors:** Hideki Komagata, Takaya Ichimura, Yasuka Matsuta, Masahiro Ishikawa, Kazuma Shinoda, Naoki Kobayashi, Atsushi Sasaki

**Affiliations:** a Saitama Medical University, Faculty of Health and Medical Care, Saitama, Japan; b Saitama Medical University, Department of Pathology, Saitama, Japan; c Japanese Red Cross Saitama Hospital, Saitama, Japan; d Utsunomiya University, Graduate School of Engineering, Tochigi, Japan

**Keywords:** cytology, feature analysis, chromatin distribution, cervical cancer, stepwise support vector machine

## Abstract

Cytology, a method of estimating cancer or cellular atypia from microscopic images of scraped specimens, is used according to the pathologist’s experience to diagnose cases based on the degree of structural changes and atypia. Several methods of cell feature quantification, including nuclear size, nuclear shape, cytoplasm size, and chromatin texture, have been studied. We focus on chromatin distribution in the cell nucleus and propose new feature values that indicate the chromatin complexity, spreading, and bias, including convex hull ratio on multiple binary images, intensity distribution from the gravity center, and tangential component intensity and texture biases. The characteristics and cellular classification accuracies of the proposed features were verified through experiments using cervical smear samples, for which clear nuclear morphologic diagnostic criteria are available. In this experiment, we also used a stepwise support vector machine to create a machine learning model and a cross-validation algorithm with which to derive identification accuracy. Our results demonstrate the effectiveness of our proposed feature values.

## 1 Introduction

Despite recent improvements in our understanding of molecular changes in cancer cells, it remains difficult to diagnose cancer using biologic methods. Some biologic methods, such as fluorescent *in situ* hybridization (FISH) for detecting chromosomal translocation and polymerase chain reaction (PCR) for detecting cell clonality, are sometimes used to assist the cancer diagnosis; however, cell clonality and chromosomal translocation are not limited features of the cancer cells.^1^^–^^4^ Cancer is always diagnosed by pathologists via light microscopic evaluations of histological or cytological samples. These diagnoses are based on the degrees of structural and cellular atypia.^5^ Among the many morphological changes occurring in cancer cells, nuclear atypia is one of the most important. Nuclear atypia refers to an abnormal cell nuclear appearance and includes changes in the nuclear size and shape, numbers and sizes of nucleoli, and chromatin texture. However, pathological evaluations of nuclear atypia may display a lack of consistency owing to variability depending on the cytologists.^6^ In fact, cytological and histological diagnostic reproducibility and accuracy are problematic for some cell types (e.g., erythroblasts).^7^ Therefore, quantitative feature analysis of nuclear atypia can enhance the cytologist’s assessment accuracy.

Conversely, to prevent overlook, cell diagnostic support systems that continuously process the extraction of cell regions (segmentation), feature extraction, and cell-type prediction (classification by a machine learning method) have also been studied.^8^^–^^12^ Because improvement in the accuracy of the system is required, addition of new features is effective. With respect to the feature extraction aspect of the system, several methods have been proposed, including quantifications of nuclear size, shape, and brightness;^13^ Haralick^14^ or run-length^15^ analysis of chromatin texture;^10^^,^^16^^–^^18^ cell nuclear contour complexity (CC);^19^ and radial distribution (RD) value.^20^ Although the CC value quantified the complexity of chromatin distribution in large areas, it did not consider chromatin distribution in small areas. In addition, the RD value focused on the deviation of the chromatin distribution only in the radial direction.

In this study, we aim to propose useful new features to facilitate judgments by cytologists and increase the accuracy of cell diagnostic support systems. Specifically, we propose three kinds of new feature values quantified by a complexity value considering chromatin distribution with small areas, a spreading value for chromatin distribution, and a tangential bias (TB) value for chromatin distribution. The proposed feature values include convex hull ratios on multiple binary images, intensity distribution from the gravity center, and tangential component intensity and texture bias. The characteristics of these proposed feature values are verified through experiments using cervical smear samples. In particular, the nuclear morphology-based diagnostic criteria for cervical cytology are clear, and interobserver differences in assessments are small.^7^ In these experiments, we compare our proposed feature values with the annotations classified by pathologists according to the Bethesda system.^21^

Thereafter, we examine the effectiveness of our proposed feature values using an analysis of variance (ANOVA) and a cross validation (CV)^22^ of models generated via a machine learning method. For machine learning, we use a support vector machine (SVM)^23^^,^^24^ that is extended to a multiclass classification using a one-versus-one method^25^ and perform variable selection using a stepwise (floating) method.^26^

## 2 Feature Extraction of Cervical Cytology Image

### 2.1 Extraction of Cell Nuclear Area

We used cervical smear samples collected at the Department of Gynecologic Oncology, Saitama Medical University International Medical Center. These samples were applied to slides, fixed with 95% alcohol, and subjected to Papanicolaou staining. Squamous cells in these samples were observed and imaged at 1000× (10× eyepiece and 100× objective lenses) magnification with an optical microscope (AXIO imager A1; Carl Zeiss Ltd., Oberkochen, Germany) attached to a cooled charge-coupled device camera (256 shades of gray) and three transmission filters of red, green, and blue. In each shooting, exposure time and white balance were fixed. In this study, we targeted squamous cells only.

**Fig. 1:**
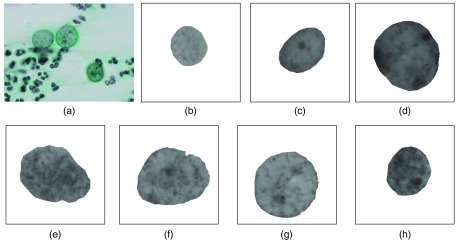
Examples of cervical cytological images. (a) Original image and (b)–(f) mask extraction image of each cell nuclei: NOR, MET, REG, LSIL, HSIL, CIS, and SCC, respectively.

Cells were estimated from these images by a pathologist and two cytotechnologists according to the Bethesda system.^21^ The classifications are as follows: negative for intraepithelial lesion or malignancy (NILM), atypical squamous cells of undetermined significance (ASC-US), low-grade squamous intraepithelial lesion (LSIL), high-grade squamous intraepithelial lesion (HSIL), atypical squamous cells but cannot exclude HSIL (ASC-H), and squamous cell carcinoma (SCC). ASC-US and ASC-H, respectively, represent intermediate classifications between NILM and LSIL and between LSIL and HSIL. In this paper, we avoided these intermediate classifications and used only typical cells (NILM, LSIL, HSIL, and SCC). In addition, we divided NILM cases into three types: normal cell (NOR), metaplastic cell (MET), and regenerative cell (REG). MET and REG include reactive nuclear atypia, which is occasionally difficult to discriminate from neoplastic nuclear atypia [LSIL, HSIL, carcinoma *in situ* (CIS), and SCC]. Therefore, it is important to classify these cell types using image-based characterization system. Although several detail studies included the image-based cell classification system, few were investigated using the detailed NILM classification.^16^^,^^20^^,^^27^ We also divide SCC cases as CIS and SCC because cytologists usually distinguish these two categories. We, therefore, evaluated seven types of cells: NOR, MET, REG, LSIL, HSIL, CIS, and SCC. Figure 1(a) shows representative images including CIS cells.

Subsequently, we manually extracted cell nuclear regions from images to generate masking images and transformed these from RGB color to gray-scale according to the Y value in the YCbCr color system. Figures 1(b)–1(h), respectively, show examples of the gray-scale masking images of NOR, MET, REG, LSIL, HSIL, CIS, and SCC. Although automatic methods for extracting cell and cell nuclear regions have been proposed,^8^ these are not completely accurate. We, therefore, manually extracted the studied cell nuclear regions.

### 2.2 Conventional Feature Values

Previous studies of the feature quantification of cervical cytology images have used feature values related to the cell nuclear size and shape.^8^^,^^13^^,^^20^^,^^28^ We also use eight feature values (nuclear area $f_{1}$, nuclear perimeter $f_{2}$, nuclear longest diameter $f_{3}$, nuclear shortest diameter $f_{4}$, nuclear convex hull area $f_{5}$, nuclear convex hull perimeter $f_{6}$, nuclear circularity $f_{7}$, and nuclear extension $f_{8}$). In this paper, convex hull refers to a convex line (i.e., shape of a rubber band) surrounding the nuclear outline. $f_{7}$ and $f_{8}$ are represented by the following equations:

$$f_{7}=\frac{4\pi f_{1}}{f_{2}^{2}},$$ (1)

$$f_{8}=\frac{f_{3}}{f_{4}}.$$ (2)

Murata et al.^16^ used other nuclear shape values to evaluate images of thyroid tumor cytology specimens. These values can be expressed using the following equations:

$$f_{9}=\frac{\pi f_{3}^{2}}{4f_{1}},$$ (3)

$$f_{10}=\frac{f_{2}}{f_{6}},$$ (4)

where $f_{9}$ represents the roundness (with numerical value decreasing with rounded) and $f_{10}$ represents the convex hull ratio of the outer shape of the nucleus. These values are calculated for the cell nuclear regions extracted manually as in Sec. 2.1.

Feature values have also been proposed for chromatin distribution in the cell nucleus, including the average value, the number of maximum value, and the number of minimum value of the image pixel intensities in nuclear regions.^8^^,^^13^^,^^28^ In addition, the RD value^20^ represents the difference in average intensity between the center and periphery of the cell nucleus and has been suggested. Regarding the gray-scale masking images described in Sec. 2.1, we also use these four chromatin distribution feature values, represented as $f_{11}$, $f_{12}$, $f_{13}$, and $f_{14}$, respectively. Murata et al. also used skewness, kurtosis, the coefficient of variation, and the upper 20 percentile ratio of the intensity histogram. The coefficient of variation is defined as the ratio between the average value and standard deviation. We also use these values and denote as $f_{15}$, $f_{16}$, $f_{17}$, and $f_{18}$, respectively.

Murata et al.^10^^,^^16^^–^^18^ used 15 texture feature values, in which 10 are Haralick feature values^14^ calculated using cooccurrence matrices and 5 are run-length feature values^15^ calculated using run-length matrices. Both matrices are calculated from gray-scale intensities within cell nuclei. The 10 Haralick feature values are contrast ($f_{19}$, contrast of intensity), energy ($f_{20}$, uniformity of intensity and texture), correlation ($f_{21}$, correlation of intensity and texture), variance ($f_{22}$, variance of intensity), entropy ($f_{23}$, diversity of intensity and texture), sum variance ($f_{24}$, contrast of intensity and texture), sum entropy ($f_{25}$, diversity of intensity), difference variance ($f_{26}$, variance of texture), difference entropy ($f_{27}$, diversity of texture), and inverse difference moment ($f_{28}$, homogeneity). The five run-length feature values are gray level nonuniformity ($f_{29}$, ununiformity of intensity), run percentage ($f_{30}$, ununiformity of intensity and texture), short run emphasis ($f_{31}$, magnitude of high frequency), long run emphasis ($f_{32}$, magnitude of low frequency), and run-length nonuniformity ($f_{33}$, nonuniformity of texture).

The cooccurrence matrix represents the appearance frequency P [=P(i,j)] of pixel intensities on a gray-scale image, where i is the intensity of a pixel of interest A and j is the intensity of a pixel B near A. We used gray-scale images of 256 gradations, and the matrix size became 256×256. Multiple cooccurrence matrices can be generated using the differences in distance values (r) and argument values (θ) between A and B. We used four types each of r (r=1, 2, 4, and 8 pixels) and four types each of θ (θ=0  deg, 45 deg, 90 deg, and 135 deg), generated 16 cooccurrence matrices, and used the averages of Haralick feature values calculated by their 16 matrix as $f_{19}$ to $f_{28}$.

The run-length matrix represents the appearance frequency R [=R(i,l)] of run l in the pixel of interest i. Run indicates the number of consecutive identical intensity values in the scanning direction θ. The intensity gradient is frequently subjected to quantization before creating a run-length matrix. We, therefore, used four types each of θ (θ=0  deg, 45 deg, 90 deg, and 135 deg) and four type each of quantization values (gradations 256, 16, 4, and 2), generated 16 run-length matrix, and used the averages of 5 run-length features calculated by their 16 run-length matrix as $f_{29}$ to $f_{33}$.

Furthermore, Kiyuna et al.^19^ previously quantified the complexity of chromatin distribution on a nuclear image from mammary gland cells as a CC value and a fractal feature. These features are represented as $f_{34}$ and $f_{35}$ in the following equations:

$$f_{34}=\underset{i}{\sum }\{\frac{L(i)}{f_{2}}-1[L(i)>f_{2}]\},$$ (5)

$$f_{35}=\max\{D(i)\}.$$ (6)

These values were calculated using a binarization of the cytology image, where i represents the intensity threshold for binarizing, and L(i) and D(i) represent the contour perimeter and a fractal dimension of the image binarized by threshold i, respectively. The values $f_{34}$ and $f_{35}$ increase as the chromatin distribution complexity increases. We used the box counting method to calculate the fractal dimension.

Another four feature values had been proposed by Haralick: sum average ($f_{36}$, average of intensity), information measures of correlation 1 ($f_{37}$, uniformity of texture), information measures of correlation 2 ($f_{38}$, diversity of texture), and maximal correlation coefficient ($f_{39}$, uniformity of intensity and texture). These features can be expressed using the following equations:

$$f_{36}=\overset{510}{\underset{k=0}{\sum }}\{k\overset{255}{\underset{i=0}{\sum }}\overset{255}{\underset{j=0}{\sum }}P(i,j)(k=i+j)\},$$ (7)

$$f_{37}=\frac{\mathrm{MI}}{-\max\{H(\mathrm{PJ}),H(P^{⊤}J)\}},$$ (8)

$$f_{38}=\sqrt{1-\exp\{-2[\hat{H}({\mathrm{PJJ}}^{⊤}P)-\hat{H}(P)]\}},$$ (9)

$$f_{39}=\sqrt{S(Q)}\;\text{where}\;Q(i,j)=\underset{k}{\sum }\frac{P(i,k)P(j,k)}{\mathrm{PJ}(i)\cdot P^{⊤}J(k)},$$ (10)

where MI is the mutual information of P, J is a vector for which all elements =1, H is an entropy function, $\hat{H}$ is a joint entropy function, and S is a function used to calculate a second eigenvalue.

We designed feature values $f_{1–39}$ as conventional feature (Cf) values.

### 2.3 Proposed Feature Values

#### 2.3.1 Convex hull contour complexity values (Pf.1)

Kiyuna et al.^19^ quantified the complexity of chromatin distribution using feature $f_{34}$. However, this feature is not counted if the perimeter L(i) is less than $f_{2}$; in other words, $f_{34}$ does not consider the chromatin complexities in small regions. Therefore, we previously proposed the following feature value F^29^

$$F=\underset{i}{\sum }\{1[\frac{L(i)}{L_{C}(i)}\geq 1.2]\},$$ (11)

where $L_{C}(i)$ and $\frac{L(i)}{L_{C}(i)}$ represent the convex hull perimeter and convex hull ratio, respectively, of an image binarized using threshold i, and the expression in Σ represents the indicator function. F is a variable that counts the number of binarization threshold values i, in which the convex hull ratio is 1.2 or more. The value of F increases with chromatin distribution complexity such that F is counted even in small chromatin regions with sufficient complexity. However, F had a large correlation with $f_{10}$, which is the convex hull ratio of the outer shape of the nucleus.

We, therefore, use the following $L_{r}(i)$, which is a chromatin distribution complexity divided by $f_{10}$, and propose following new feature values $f_{40}$ to $f_{43}$

$$L_{r}(i)=\frac{L(i)}{L_{C}(i)\cdot f_{10}},$$ (12)

$$f_{40}=\sum_{i}\{L_{r}(i)-1[L_{r}(i)\geq 1]\},$$ (13)

$$f_{41}=\sum_{i}\{1[L_{r}(i)\geq 1.1]\},$$ (14)

$$f_{42}=\sum_{i}\{1[L_{r}(i)\geq 1.2]\},$$ (15)

$$f_{43}=\sum_{i}\{1[L_{r}(i)\geq 1.3]\}.$$ (16)

**Fig. 2:**
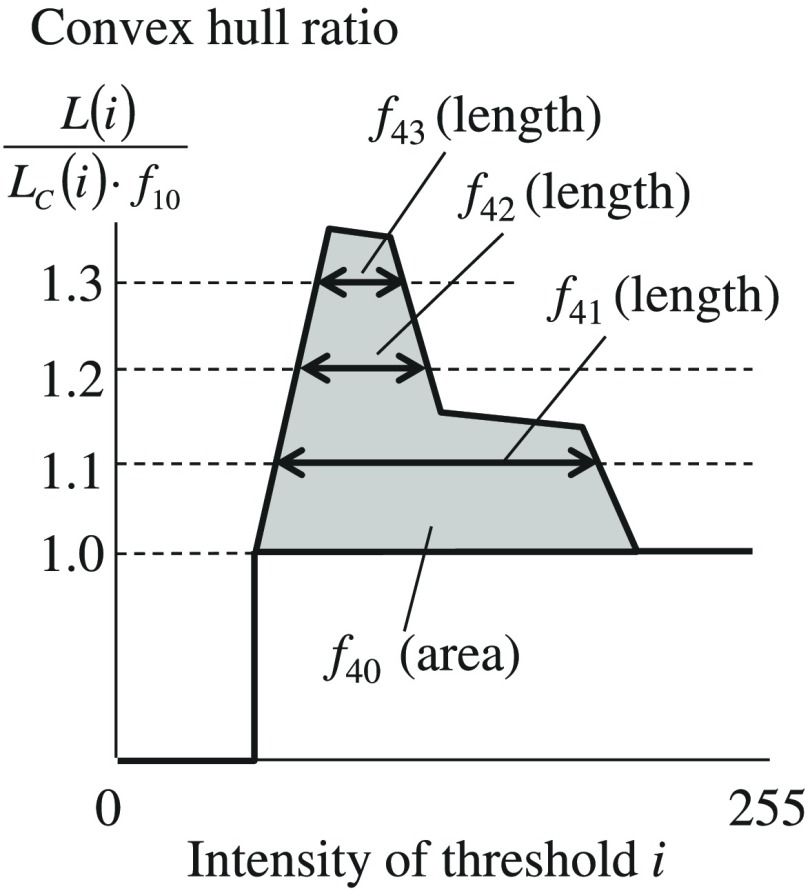
Convex hull contour complexity values.

$f_{40}$ is shown as the fill area in Fig. 2 for which the horizontal axis is the intensity threshold for binarization and the vertical axis is $L_{r}(i)$. $f_{41}$, $f_{42}$, and $f_{43}$ are shown as intensity widths of the graph when $L_{r}(i)\geq 1.1$, 1.2, and 1.3 in Fig. 2. For a more detailed representation of the shape of the graph shown in Fig. 2, multiple feature values are used. We name values of $f_{41}$, $f_{42}$, and $f_{43}$ as convex hull (CH) CC, convex hull intensity-width 1.1 (CW1.1), CW1.2, and CW1.3, respectively. We designed these four features $f_{40–43}$ as proposal feature values 1 (Pf.1).

#### 2.3.2 Chromatin distribution spreading value (Pf.2)

We also propose a method for quantifying the chromatin distribution spreading (CDS) value. First, we create an intensity histogram using the gray-scale intensity set from the cell nucleus on the input image and obtain a threshold $T_{h}$ via a linear discriminant analysis^30^ of the histogram. $T_{h}$ is the threshold used to distinguish dark-stained (i.e., assumed chromatin) and light-stained regions (i.e., nonchromatin). Here, a coordinate on the input image is designed as Z (Z =[x,y]), and the 256-level gray-scale intensity of Z is designated as I(Z). Next, a chromatin image is generated by replacing the I(Z) of all pixels in the image with $I^{\prime }(Z)$, calculated using the following equation:

$$I^{\prime }(Z)=\{\begin{array}{ll} 255-I(Z)-T_{h}, & \left[I(Z)+T_{h}<255\right]\\ 0, & \left(\mathrm{otherwise}\right) \end{array}.$$ (17)

**Fig. 3:**
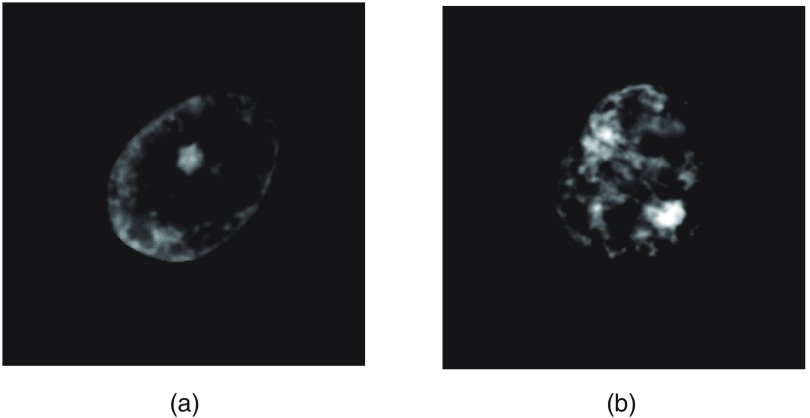
Chromatin image. (a) and (b) Image calculated from Fig. 1(c) and 1(h), respectively.

$I^{\prime }(Z)$ is a value obtained by inverting the image negative and positive [255−I(Z)] and subtracting the bias value $T_{h}$. As a result, high-density stained pixels such as nucleoli and chromatin appear as high values. Figures 3(a) and 3(b) show representative chromatin images based on those in Figs. 1(c) and 1(h), respectively, and show at fivefold intensity to enhance visualization.

Next, the center of a gravity of the chromatin region G is obtained using the following equation:

$$G=(\frac{\underset{x}{\sum }\underset{y}{\sum }xI^{\prime }(Z)}{\underset{x}{\sum }\underset{y}{\sum }I^{\prime }(Z)},\frac{\underset{x}{\sum }\underset{y}{\sum }yI^{\prime }(Z)}{\underset{x}{\sum }\underset{y}{\sum }I^{\prime }(Z)}).$$ (18)

Finally, we used G to obtain the CDS value, denoted as $f_{44}$ in the following equation:

$$f_{44}=\sqrt{\frac{1}{f_{1}}\underset{x}{\sum }\underset{y}{\sum }\{{\left|\frac{G-Z}{f_{3}}\right|}^{2}I^{\prime }(Z)\}}.$$ (19)

We designed this feature $f_{44}$ as Pf.2.

#### 2.3.3 Tangential bias values of chromatin distribution (Pf.3)

Cytologically, a biased chromatin texture distribution is an important atypical cell nuclear feature. Jingu et al.^20^ proposed the RD value, which represents radial bias in the intensity of chromatin distribution but did not consider the tangential direction. Therefore, we previously proposed a feature value E calculated by the following process and Eq. (20):^31^

i. Extraction of the outer shape of the cell nucleus and fitting to an ellipse.
ii. Calculation of the center, short axis, and long axis of the ellipse.
iii. Euclidean transformation of the input nuclear image such that the short axis, long axis, and center of the ellipse become the new X-axis, Y-axis, and origin, respectively.
iv. Creation of four images by cutting the transformed image at the X- and Y-axes.
v. Calculation of some chromatin distribution feature values $f_{t}(t\in \{11,12,\mathrm{and}\;18\;\mathrm{to}\;44\}$) on each of the four images to yield $f_{t,1},f_{t,2},f_{t,3}$, and, $f_{t,4}$

$$E=\mathrm{SD}(f_{t,1},f_{t,2},f_{t,3},f_{t,4}),$$ (20)

where SD is a function used to calculate the standard deviation. However, since the standard deviation is easily influenced by the magnitude of each feature values, we use coefficient of variation instead of the standard deviation as shown in the following equation:

$$g_{t}=|\frac{\mathrm{SD}(f_{t,1},f_{t,2},f_{t,3},f_{t,4})}{\mathrm{mean}(f_{t,1},f_{t,2},f_{t,3},f_{t,4})}|,$$ (21)

where mean is a function used to calculate the mean.

In this paper, we propose TB values $f_{45}$ and $f_{46}$ of chromatin distribution using $g_{t}$. TB values, denoted as $f_{45}$ and $f_{46}$, are determined experimentally in Sec. 3.

## 3 Evaluation of the Tangential Bias Values for (Pf.3)

To experimentally examine TB in the chromatin distribution, we created 633 masking images from 633 cervical smear samples according to the method described in Sec. 2.1. Table 1 shows the numbers of cell nuclei, slides, and patients for each cell classification. We prepared one slide per patient and took images of a single cell type for each slide. These samples included 164, 86, 74, 36, 155, 84, and 34 cases of NOR, MET, REG, LSIL, HSIL, CIS, and SCC, respectively. We then calculated the $g_{t}$ of each masking image as described in Sec. 2.3.3. Thereafter, the $g_{t}$ values were linearly normalized as $g_{t}^{\prime }$, thus converting the maximum and minimum values of each $g_{t}$ to 1 and 0. Accordingly, $g_{t}^{\prime }$ can be represented as follows:

$$g_{t}^{\prime }=\frac{g_{t}}{\max(g_{t})-\min(g_{t})}.$$ (22)

**Fig. 4:**
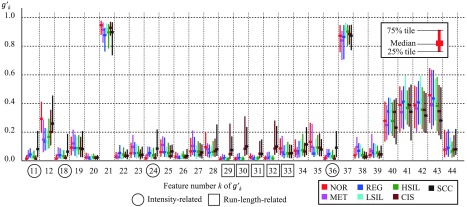
Feature bias values $g_{t}^{\prime }$.

Figure 4 shows an experimental result from a calculation using 75% tiles, median values, and 25% tiles of the $g_{t}^{\prime }$ for each cell type. The horizontal axis indicates the feature number, and colors indicate the types of cells annotated by pathologists. The circles in Fig. 4 indicate the feature values related to intensity, which had notably high values ($g_{11}^{\prime }$, $g_{18}^{\prime }$, $g_{24}^{\prime }$, $g_{36}^{\prime }$) in SCC. The squares in Fig. 4 indicate the run-length feature values, which are included among the texture features; here, $g_{29}^{\prime }$ to $g_{32}^{\prime }$ were explicitly high for both CIS and SCC. These values could, therefore, be useful for cellular classification.

**Table 1:** Numbers of cell nuclei, slides, and patients.

	NOR	MET	REG	LSIL	HSIL	CIS	SCC	Total
Cell nuclei	164	86	74	36	155	84	34	633
Slides	6	6	6	3	3	5	3	32
Patients	6	6	6	3	3	5	3	32

We, therefore, propose two TB values $f_{45}$ and $f_{46}$, as shown in the following equations:

$$f_{45}=\frac{g_{11}+g_{18}+g_{24}+g_{36}}{4},$$ (23)

$$f_{46}=\frac{g_{29}+g_{30}+g_{31}+g_{32}}{4},$$ (24)

where $f_{45}$ and $f_{46}$ represent the biases of intensity distribution and texture distribution, respectively. We designed these two features $f_{45}$ and $f_{46}$ as Pf.3. In addition, we designed the entire set of features $f_{1–46}$ as proposal feature values of all (Pf.A).

To minimize the variability of staining, we used the same staining machine and protocol and omitted poor samples (such as dried samples). To minimize the influence of intensity fluctuation during scanning, we photographed each slide with fixed exposure time and white balance. However, small variations in staining due to the different conditions of the samples cannot be excluded. Feature values related to intensity ($f_{11,18,24,36}$) may be influenced by these effects to a substantially greater degree than feature values related to shape and texture. Note that $f_{45}$ is a feature value derived from other intensity-related feature values; however, the effects of sample condition are smaller than on other intensity-related feature values ($f_{11,18,24,36}$), because $f_{45}$ uses the coefficient of variation.

## 4 Verification of the Characteristics of the Proposed Feature Values

### 4.1 Comparative Experimental Results and Discussion Between Cell Types

**Fig. 5:**
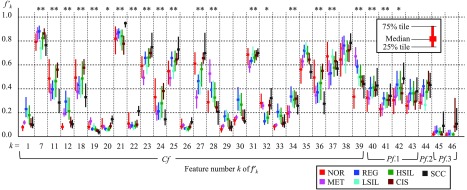
Experimental results from a feature value analysis of seven cell types.

To verify the characteristics of our proposed feature values, we calculated feature values $f_{1–46}$ from 633 masking images of cervical smear samples described in Sec. 3. Thereafter, features $f_{1–46}$ were linearly normalized to yield $f_{1–46}^{\prime }$ such that the maximum and minimum values of each feature became 1 and 0. Figure 5 shows an experimental result calculated using the 75% tiles, median values, and 25% tiles of features $f_{1–46}^{\prime }$ for each cell types. However, we note that some Cf values were omitted.

Figure 5 shows many differences in feature values among NOR, NET, and REG, which we classified as NILM. In particular, REG had a large area ($f_{1}^{\prime }$ was high), and MET exhibited high texture homogeneity ($f_{28}^{\prime }$ and $f_{39}^{\prime }$ were high, whereas $f_{23}^{\prime }$ and $f_{27}^{\prime }$ were low). All proposed value $f_{40–46}^{\prime }$ were small for NOR and large for SCC. In particular, $f_{44}^{\prime }$ and $f_{46}^{\prime }$ were also large for CIS. Cancer cells possess a chromatin structure that differs from the normal structure.^5^ These results suggest that our proposed method represents the features of this chromatin structure.

In addition, we calculated the absolute $\left|\rho_{i,j}\right|$ values of the correlation coefficients between $f_{i}^{\prime }$ and $f_{j}^{\prime }$ (i∈{1,2,…,46}, j∈{1,2,…,46}) using SPSS software (IBM Corporation, Armonk, New York). $\rho_{i,j}$ is expressed by the following equation:

$$\rho_{i,j}=\frac{\mathrm{cov}(f_{i}^{\prime },f_{j}^{\prime })}{\sqrt{\mathrm{var}(f_{i}^{\prime })}\sqrt{\mathrm{var}(f_{j}^{\prime })}},$$ (25)

where cov and var represent functions for calculating the sample covariance and variance, respectively.

Table 2 shows a list of feature numbers (k) with high correlation coefficients ($|\rho_{i,j}|\geq 0.95$ or $0.95>|\rho_{i,j}|\geq 0.90$) with other features. There were strong correlations between size and shape features $f_{1–6}^{\prime }$ and the run-length features $f_{30,33}^{\prime }$. There were also strong correlations among intensity-related features $f_{11,18,24,36}^{\prime }$, all of which are Cfs. In contrast, proposed features $f_{40–46}^{\prime }$ did not show strong correlations with any other features; therefore, our proposed features are highly original. These various feature values are useful for improving the accuracy of machine-learning-based cellular classification, which we will discuss further in Sec. 5.

**Table 2:** List of feature numbers i and j with high correlation coefficient.

i	j when $|\rho_{i,j}|\geq 0.95$	j when $0.95>|\rho_{i,j}|\geq 0.90$	i	j when $|\rho_{i,j}|\geq 0.95$	j when $0.95>|\rho_{i,j}|\geq 0.90$
1	2, 4, 5, 6, 30, 33	3, 12, 29	22		20
2	1, 3, 4, 5, 6, 30, 33		23		25
3	2, 6	1, 5, 30, 33	24	11, 18, 36	
4	1, 2, 5, 6	30, 33	25		23
5	1, 2, 4, 6, 30, 33	3, 12, 29	26	20	
6	1, 2, 3, 4, 5, 30	33	27		28
8	9		28		27
9	8		29		1, 5, 33
11	18, 24, 36		30	1, 2, 5, 6, 33	3, 4
12	13	1, 5	33	1, 2, 5, 30	3, 4, 6, 29
13	12		36	11, 18, 24	
18	11, 24, 36		37		38
20	26	22	38		37

Furthermore, we used SPSS software to perform a one-dimensional ANOVA of the cellular classification corresponding to each feature value. Accordingly, all values ($f_{40–46}^{\prime }$) differed significantly (significance level: 1.0%) among the cellular classifications, and therefore, any proposed or Cf values could potentially improve the accuracy of cellular classification accuracy.

Next, we used a t-test to evaluate whether each feature value differed significantly with respect to reactive (MET and REG) and neoplastic nuclear atypia (LSIL, HSIL, CIS, and SCC). The results are shown in Fig. 5(upper): here, the * and ** symbols indicate that the corresponding feature values had significant differences at respective significance levels of 5.0% and 1.0%. This test revealed significant differences in many feature values related to chromatin distribution, including the proposed values $f_{44–46}^{\prime }$. These could, therefore, be considered useful for distinguishing between reactive and neoplastic nuclear atypia.

### 4.2 Verification of Experimental Results and Discussion of Representative Images

**Fig. 6:**
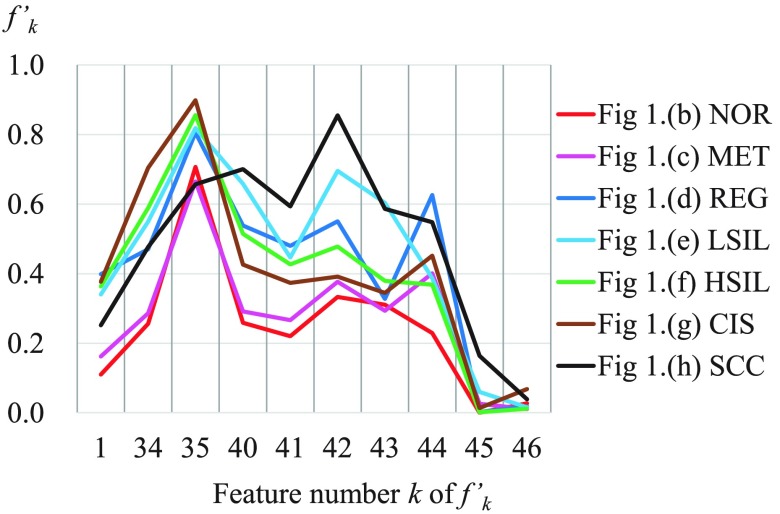
Feature values of the images in Fig. 1.

We next calculated some of the normalized feature values $f_{1,34,35,40–46}^{\prime }$ corresponding to the representative images in Figs. 1(b)–1(f). Figure 6 shows the results, with feature numbers indicated on the horizontal axis.

In Fig. 6, many values in area $f_{1}^{\prime }$, the conventional complexity values $f_{34–35}^{\prime }$, and the proposed complexity values $f_{40–44}^{\prime }$ exhibited similar tendencies; however, in the representative image of SCC, $f_{34–35}^{\prime }$ were moderate, whereas $f_{40–44}^{\prime }$ were high. In addition, the proposed value $f_{45}^{\prime }$ was also high. Although these findings are subjective, the chromatin distribution in Fig. 1(h) appears to be complex and widely spread. We consider that our proposed method reflects this trend.

## 5 Machine Learning Validation of Proposed Methods

### 5.1 Validation Method

Next, we verified the cellular classification accuracy using machine learning and a CV method.^22^ These verifications were compared among eight different models, a conventional model (Cf) and seven models combining Cf with models including our proposed values: Cf + Pf.1, Cf + Pf.2, Cf + Pf.3, Cf + Pf.1 + Pf.2, Cf + Pf.1 + Pf.3, Cf + Pf.2 + Pf.3, and Pf.A (= Cf + Pf.1 + Pf.2 + Pf.3).

For machine learning, we used the SVM;^23^^,^^24^ however, we note that this method is intended for two-class classification. We, therefore, implemented a one-versus-one method^25^ to expand the classification from two-class to multiclass using a round-robin method of classes. In addition, we selected variables for SVM using a stepwise (floating) method.^26^ In Sec. 4.1, some of the Cfs showed high correlation coefficients. If we use all of these features directly to create a model of the SVM, the accuracy of identification may decrease due to over-learning.^32^ The stepwise method we use can mitigate the reduced classification accuracy caused by over-learning, because the possibility of simultaneously selecting features exhibiting high correlation coefficients in the method is low.

Two methods could be used to combine multiclass classification and variable selection. The first involves selecting the same type of features for each comparison, and the second involves selecting different types of features for each comparison. In this paper, we used the second method, which is capable of more detailed feature selection.

**Fig. 7:**
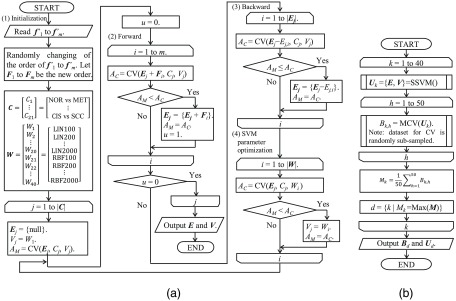
Flowchart of the (a) SSVM and (b) accuracy evaluation.

A machine learning protocol based on these methods is depicted in Fig. 7(a) as stepwise SVM (SSVM). Before performing the procedure described in Fig. 7(a), we calculated the normalized feature values of $f_{1}^{\prime }$ to $f_{m}^{\prime }$ for all 633 cervical smear samples described in Sec. 4 (NOR for 164, MET for 86, REG for 74, LSIL for 36, HSIL for 155, CIS for 84, and SCC for 34). They were a number of imbalanced samples, which can cause incorrect answer rates. We, therefore, virtually matched the sample number of each class using a oversampling method “adaptive synthetic sampling approach for imbalanced learning”^33^ to increase the number of each class up to 200 (for a total of 1400 samples) and assumed the value sets to be the feature vectors $f_{1-m}^{\prime }$, where m represents the number of features types used for calculation. For example, m becomes 39 when calculating the machine learning model Cf.

In (1) of Fig. 7(a), the order of the normalized feature values $f_{1-m}^{\prime }$ changed randomly with the initialization of some variables. We assumed the changed values to be feature vectors $F_{1-m}$. Here, C is a set representing the round-robin selection of seven classes, W is a set representing the kernel functions and cost parameters among the SVM parameters, $E_{j}=\{E_{j,1},\ldots ,E_{j,i},\ldots \}$ is a set of feature vectors $F_{1-m}$ selected for model $C_{j}$, $V_{j}$ is the SVM parameter selected for model $C_{j}$, $\mathrm{CV}(E_{j},C_{j},V_{j})$ is a function used to calculate the accuracy rate from the CV of the SVM, $A_{M}$ is the maximum accuracy rate calculated by the CV, and u is an updated flag of the maximum accuracy rate. In addition, LIN, RBF, and the numeric values of the elements of W in Fig. 7(a), respectively, represent a linear function of the kernel, a radial basis function of the kernel, and the cost parameters included among the SVM parameters. In this paper, we used 10-fold as the number of CV divisions.

In Fig. 7(a), (2), (3), and (4), respectively, represent procedures involving forward feature selection, backward feature selection, and SVM parameter optimization. These values were calculated based on the CV of the SVM. Here, $A_{C}$ is an accuracy rate calculated by the CV, E is a power set of $E_{j}$, and V is a power set of $V_{j}$. Feature selection was implemented by calculating these procedures until the maximum accuracy rate was no longer updated, and SSVM was implemented by calculating procedures using the round-robin selection of seven classes.

Figure 7(b) shows an accuracy evaluation procedure based on the SSVM, where $U_{k}$ is a set of E and V, SSVM is a function used to calculate the procedure in Fig. 7(a), MCV is a function used to calculate the accuracy rate from the multiclass CV according to the one-versus-one method, $B_{k,h}$ is an accuracy rate obtained from the MCV, $M_{k}$ is a mean accuracy rate obtained by repeatedly (50×) calculating the MCV, M is a set of $M_{k}$ obtained by repeating these procedures, and d is an index number of the maximum value of M. SSVM is likely to fall into a local solution, and $U_{k}$ is not necessarily the optimum value when obtained from the calculation of a single SSVM. In other words, the selected features and accuracy may be affected by the order of the initial data set. We, therefore, randomly exchanged data sets and extracted the optimum value by repeating the SSVM from k=1 to 40 to eliminate the fall into a local solution as much as possible.

Finally, the optimum accuracy rate set $B_{d}=\{B_{d,1},\ldots ,B_{d,k},\ldots ,B_{d,50}\}$ and parameter set $U_{d}$ were outputted, and the results of eight classification models (Cf, Cf + Pf.1, Cf + Pf.2, Cf + Pf.3, Cf + Pf.1 + Pf.2, Cf + Pf.1 + Pf.3, Cf + Pf.2 + Pf.3, and Pf.A) were compared.

### 5.2 Validation Results and Discussion

We calculated the averages (Ave.) and standard deviations (SD.) of the accuracy rate set $B_{d}$ of the eight classification models, using the validation method shown in Sec. 5.1. Table 3 presents the results of a comparison of these values, as well as the Dunnett’s test (D-test) results for each model. Here, D-test 1 represents the D-test results of comparisons between each models and Cf, D-test 2 represents the D-test results of comparisons between each model and Pf.A, and ** indicates a significant difference (significance level = 5.0%). D-test is a multiple comparison, many-to-one procedure (i.e., compares each of many treatment groups with one control group) and is used to verify differences between the average values from each group.^34^^,^^35^ We used SPSS software to perform this procedure.

**Table 3:** Experimental accuracy rates for each model.

	Cf	Cf + Pf.1	Cf + Pf.2	Cf + Pf.3	Cf + Pf.1+ Pf.2	Cf + Pf.1+ Pf.3	Cf + Pf.2+ Pf.3	Pf.A
Avg. (%)	86.80	87.52	86.81	87.25	87.74	88.22	87.46	88.44
SD. (%)	0.88	0.96	1.02	1.02	0.89	0.88	0.87	1.05
D-test 1	—	**		**	**	**	**	**
D-test 2	**	**	**	**	**	**	**	—

Ave. and SD., respectively, represent the average and standard deviation of the accuracy rate set $B_{d}$ for each of eight classifications. D-tests 1 and 2 represent comparisons with Cf and Pf.A, respectively. ** represents a significant difference at a level of 5.0%.

The average accuracy rates of all proposed models except Cf + Pf.2 were higher than the conventional model (Cf) and exhibited statistically significant differences from Cf by the D-test. Therefore, our proposed models Pf.1 and Pf.3 (features $f_{40–43,45–46}$) are useful features for cervical cell classification by machine learning. Although there was no significant difference between Cf + Pf.2 and Cf, there was a significant difference between Pf.A and Cf + Pf.1 + Pf.3. Therefore, our Pf.2 (feature $f_{44}$) is also a useful feature for cervical cell classification. These results show the usefulness of incorporating our features into the diagnostic support system of the cytology. In addition, these results indicate that our features are different from the Cfs; therefore, our features have the possibility to be useful features in cell diagnosis by the cytologist.

**Fig. 8:**
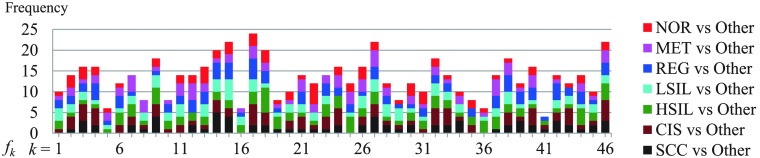
Frequency of selected features in the Pf.A model.

As shown in 5.1, to extend the SVM to a multiclass classification of seven classes, we performed the SVM 21 times in the round-robin selection format; in other words, we obtained 21 selected feature sets E ($E\subset U_{d}$) to create a single machine learning model. We, therefore, extracted the 21 selected feature sets E of Pf.A, which had the highest accuracy rate and calculated the frequencies as shown in Fig. 8 (cumulative bar chart). Red, magenta, blue, cyan, green, brown, and black colors indicate selected features from comparisons related to NOR, MET, REG, LSIL, HSIL, CIS, and SCC, respectively. Based on Fig. 8, the selection of all proposal features indicates that all contributed to improve the classification accuracy.

## 6 Conclusion

Although cytology is a useful diagnostic tool for cervical and other conditions, it is generally used empirically. In this paper, we aimed to quantify the cell nuclear morphologies often used in cytologic analyses and proposed three new types of feature values: Pf.1, Pf.2, and Pf.3. Pf.1 includes CH CC values that represent the complexity of chromatin distribution within the cell nucleus. Pf.2 is the CDS, which represent intensity spreading from the gravity center in the chromatin region. Pf.3 is the TB values of chromatin distribution, which were calculated using the coefficient of variation for the intensities and run-length texture values of nuclear images that had been divided into four images based on the center of a fitted ellipse.

We used three methods to verify these proposal feature values. All methods used 633 images of nuclei obtained from the cervical cytology specimens of 32 patients and cell type information (NOR, MET, REG, LSIL, HSIL, CIS, or SCC) that had been annotated by a pathologist and two cytotechnologists.

The first method used an ANOVA to determine whether the proposal feature values differed among the seven classes. We found that all proposal values differed significantly at a 1.0% significance level, indicating the usefulness of these proposed feature values for cervical cytology. The second method used the t-test to determine differences in our proposed feature values between reactive (MET and REG) and neoplastic nuclear atypia (LSIL, HSIL, CIS, and SCC). We found that our proposed values CH, CW1.1, and CW1.2 differed at a 5.0% significance level, indicating their usefulness as distinguishing factors.

The third method determined whether the classification accuracy among the seven classes improved when multiple sets of feature values were combined through SSVM and a machine learning technique with a variable selection function. We calculated the accuracy of these finding using the CV method, calculated accuracy distribution using several repeats, and verified effectiveness using D-tests. We used eight different models, the conventional model (Cf) and seven models combining Cf with proposed models: Cf + Pf.1, Cf + Pf.2, Cf + Pf.3, Cf + Pf.1 + Pf.2, Cf + Pf.1 + Pf.3, Cf + Pf.2 + Pf.3, and Pf.A (= Cf + Pf.1 + Pf.2 + Pf.3). Accordingly, average accuracy rates of all proposed models except Cf + Pf.*2* were higher than the conventional model (Cf) and exhibited statistically significant differences from Cf by D-test. This indicates that Pf.1 and Pf.3 are useful for cervical cell classification by machine learning. Although there was no significant difference between Cf + Pf.2 and Cf, there was a significant difference between Pf.A and Cf + Pf.1 + Pf.3; therefore, Pf.2 is also useful for cervical cell classification. The model created via SSVM selected all proposed feature values, and the results indicated that all proposed features contributed to the improved classification accuracy.

We proposed features reflecting the complexity, spreading, and bias of the chromatin distribution and showed that classification accuracy rates were increased by combining our features with Cfs. These results show the usefulness of incorporating our features into a diagnostic support system for cytology. In addition, these results indicate that our features are different from the Cfs; therefore, our features have the possibility to be useful features in cell diagnosis by the cytologist.

Meanwhile, since the evaluation of the usefulness of individual feature values in actual clinical diagnosis was not conducted, continuing studies are necessary to evaluate the usefulness in clinical practice. In addition, although we focused on the cell nucleus, the cytoplasm is also an important indicator. In the future, we aim to quantify the features of the cell cytoplasm and continue studies to evaluate the usefulness in clinical practice.

## References

[1] Janz S. Potter M. Rabkin C. S., “Lymphoma- and leukemia-associated chromosomal translocations in healthy individuals,” Genes Chromosomes Cancer 36(3), 211–223 (2003). 10.1002/(ISSN)1098-2264 12557221

[r2] Nambiar M. Raghavan S. C., “Chromosomal translocations among the healthy human population: implications in oncogenesis,” Cell. Mol. Life Sci. 70(8), 1381–1392 (2013). CMLSFI 1420-9071 10.1007/s00018-012-1135-x 22948164 PMC11113647

[r3] Posnett D. N. et al., “Clonal populations of T cells in normal elderly humans: the T cell equivalent to ‘benign monoclonal gammapathy’,” J. Exp. Med. 179(2), 609–618 (1994). JEMEAV 0022-1007 10.1084/jem.179.2.609 8294871 PMC2191374

[4] Iijima T. Inadome Y. Noguchi M., “Clonal proliferation of B lymphocytes in the germinal centers of human reactive lymph nodes: possibility of overdiagnosis of B cell clonal proliferation,” Diagn. Mol. Pathol. 9(3), 132–136 (2000). DMPAES 1052-9551 10.1097/00019606-200009000-00002 10976719

[5] Zink D. Fischer A. H. Nickerson J. A., “Nuclear structure in cancer cells,” Nat. Rev. Cancer 4, 677–687 (2004). NRCAC4 1474-175X 10.1038/nrc1430 15343274

[6] DeMay R. M., “Common problems in papanicolaou smear interpretation,” Arch. Pathol. Lab. Med. 121(3), 229–238 (1997). 9111106

[7] Parmentier S. et al., “Assessment of dysplastic hematopoiesis: lessons from healthy bone marrow donors,” Haematologica 97(5), 723–730 (2012). HAEMAX 0390-6078 10.3324/haematol.2011.056879 22180437 PMC3342975

[8] Duanggate C. Uyyanonvara B. Koanantakul T., “A review of image analysis and pattern classification techniques for automatic pap smear screening process,” in Int. Conf. on Embedded Systems and Intelligent Technology, pp. 212–217 (2008).

[r9] Isa N. A. M., “Automated edge detection technique for pap smear images using moving k-means clustering and modified seed based region growing algorithm,” Int. J. Comput. Internet Manage. 13(3), 45–59 (2005).

[r10] Chen Y. F. et al., “Semi-automatic segmentation and classification of pap smear cells,” IEEE J. Biomed. Health Inform. 18(1), 94–108 (2014). 10.1109/JBHI.2013.2250984 24403407

[r11] Watanabe S. Group T. C., “An automated apparatus for cancer prescreening: CYBEST,” Comput. Graphics Image Process. 3(4), 350–358 (1974). CGIPBG 0146-664X 10.1016/0146-664X(74)90029-X

[12] Holmquist J. et al., “Computer analysis of cervical cells automatic feature extraction and classification,” J. Histochem. Cytochem. 26(11), 1000–1017 (1978). 10.1177/26.11.569164 569164

[13] Jantzen J. Dounias G., “Analysis of pap-smear image data,” in Proc. of Nature-Inspired Smart Information Systems 2nd Annual Symp. (2006).

[14] Haralick R. M. Shanmugam K. Dinstein I., “Texture feature for image classification,” IEEE Trans. Syst., Man, Cybern. SMC-3(6), 610–621 (1973). ISYMAW 0018-9472 10.1109/TSMC.1973.4309314

[15] Galloway M. M., “Texture analysis using gray level run lengths,” Comput. Graphics Image Process. 4(2), 172–179 (1975). CGIPBG 0146-664X 10.1016/S0146-664X(75)80008-6

[16] Murata S. et al., “Morphological abstraction of thyroid tumor cell nuclei using morphometry with factor analysis,” Microsc. Res. Tech. 61(5), 457–462 (2003). MRTEEO 1059-910X 10.1002/(ISSN)1097-0029 12845572

[r17] Niwas S. I. Palanisamy P. Sujathan K., “Complex wavelet based texture features of cancer cytology images,” in 5th Int. Conf. on Industrial and Information Systems, pp. 348–353 (2010). 10.1109/ICIINFS.2010.5578679

[18] Kowal M. Filipczuk P., “Nuclei segmentation for computer-aided diagnosis of breast cancer,” Int. J. Appl. Math. Comput. Sci. 24(1), 19–31 (2014). 10.2478/amcs-2014-0002

[19] Kiyuna T. et al., “Characterization of chromatin texture by contour complexity for cancer cell classification,” in 8th IEEE Int. Conf. on BioInformatics and BioEngineering (BIBE ’08), pp. 1–6 (2008). 10.1109/BIBE.2008.4696831

[20] Jingu R. et al., “Quantitative image analysis of nuclear chromatin distribution for cytological diagnosis,” Acta Cytol. 55(5), 455–459 (2011). ACYTAN 0001-5547 10.1159/000330672 21986174

[21] Apgar B. S. Zoschnick L. Wright T. C., “The 2001 Bethesda system terminology,” Am. Fam. Physician 68(10), 1992–1998 (2003). 14655809

[22] Forsyth D. A. Ponce J., Computer Vision: A Modern Approach, Prentice Hall (2002).

[23] Cortes C. Vapnik V., “Support-vector networks,” Mach. Learn. 20, 273–297 (1995). MALEEZ 0885-6125

[24] Chang C. C. Lin C. J., “LIBSVM: a library for support vector machines,” ACM Trans. Intell. Syst. Technol. 2(3), 1–27 (2011). 10.1145/1961189

[25] Hsu C. W. Lin C. J., “A comparison of methods for multi-class support vector machines,” IEEE Trans. Neural Networks 13, 415–425 (2002). ITNNEP 1045-9227 10.1109/72.991427 18244442

[26] Wang L. et al., “A novel stepwise support vector machine (SVM) method based on optimal feature combination for predicting miRNA precursors,” Afr. J. Biotechnol. 10(74), 16720–16731 (2011).

[27] Zhao M. et al., “Feature quantification and abnormal detection on cervical squamous epithelial cells,” Comput. Math. Methods Med. 2015, 941680 (2015). 10.1155/2015/941680 25873991 PMC4385601

[28] Jantzen J. et al., “Pap-smear benchmark data for pattern classification,” in Proc. of Nature-Inspired Smart Information Systems (NISIS), pp. 1–9 (2005).

[29] Ohnuki Y. et al., “A study of a quantitative evaluation method by contour complexity of the nucleus image for cancer cell diagnosis,” in Forum on Information Technology (FIT ’13), Vol. 12, pp. 401–402 (2013).

[30] Otsu N., “A threshold selection method from gray-level histograms,” IEEE Trans. Syst., Man, Cybern. 9(1), 62–66 (1979). 10.1109/TSMC.1979.4310076

[31] Komagata H. et al., “A study of eccentric quantitation approach for chromatin distribution in cytodiagnosis,” in Media Computing Conf. 2014, R4–3 (2014).

[32] Hughes G. F., “On the mean accuracy of statistical pattern recognizers,” IEEE Trans. Inf. Theory 14, 55–63 (1968). IETTAW 0018-9448 10.1109/TIT.1968.1054102

[33] He H. et al., “ADASYN: adaptive synthetic sampling approach for imbalanced learning,” in IEEE Int. Joint Conf. on Neural Networks (2008). 10.1109/IJCNN.2008.4633969

[34] Dunnett C., “A multiple comparison procedure for comparing several treatments with a control,” J. Am. Stat. Assoc. 50, 1096–1121 (1955). 10.1080/01621459.1955.10501294

[35] Dunnett C., “New tables for multiple comparisons with a control,” Biometrics 20, 482–491 (1964). BIOMB6 0006-341X 10.2307/2528490

